# The Role and Duty of Global Surgery in Increasing Sustainability and Improving Patient Care in Low and Middle-Income Countries

**DOI:** 10.7759/cureus.30023

**Published:** 2022-10-07

**Authors:** Francis P Banhidy, Norbert F Banhidy

**Affiliations:** 1 Medicine, University College London, London, GBR; 2 Plastic and Reconstructive Surgery, Royal London Hospital, London, GBR

**Keywords:** equitable surgical care, energy reduction, waste reduction, advancing patient care, innovation, global health, low income countries, sustainability, environment, global surgery

## Abstract

Global health is one of the most pressing issues facing the 21st century. Surgery is a resource and energy-intensive healthcare activity which produces overwhelming quantities of waste. Using the 5Rs (*Reduce*, *Reuse*, *Recycle*, *Rethink,* and *Research*) provides the global surgical community with the pillars of sustainability to develop strategies that are scalable and transferable in both low and middle-income countries and their high-income counterparts.

Reducing energy consumption is necessary to achieving net zero emissions in the provision of essential healthcare. Simple, easily transferrable, high-income country (HIC) technologies can greatly reduce energy demands in low-income countries. Reusing appropriately sterilized equipment and reprocessing surgical devices leads to a reduction of costs and a significant reduction of unnecessary potentially hazardous waste. Recycling through official government-facilitated means reduces 'informal recycling' schemes, and the spread of communicable diseases whilst expectantly reducing the release of carcinogens and atmospheric greenhouse gases. Rethinking local surgical innovation and providing an ecosystem that is both ethical and sustainable, is not only beneficial from a medical perspective but allows local financial investment and feeds back into local economies. Finally, research output from low-income countries is minimal compared to the global academic output. Research from low and middle-income countries must equal research from high-income countries, thereby producing fruitful partnerships. With adequate international collaboration and awareness of the lack of necessary surgical interventions in low and middle-income countries (LMICs), global surgery has the potential to reduce the impact of surgical practice on the environment, without compromising patient safety or quality of care.

## Introduction and background

Global health is increasingly one of the most vital issues of the 21st century. Surgery, however, has long been overlooked by the greater global health landscape. It was famously described as the 'neglected stepchild of global health' [1]. This is not an unfair assessment but instead a harsh reality. Of the global burden of disease, surgical management forms 30% of the care provided to a broad range of treatable diseases. Despite these numbers, investment is preferentially directed towards disease-specific initiatives (e.g., malaria) over the horizontal and non-communicable nature of surgically managed illnesses [2,3]. Approximately 266 million operations are performed every year, and only 3.5% of these are received by the world’s poorest populations in low and middle-income countries (LMICs) [4]. Global surgery is a much sought-after, rapidly developing, multidisciplinary practice that aims to provide both improved and equitable surgical care across international health systems [5].

Global surgery is not without its limitations, and of specific interest is the environmental impact of surgery. The United States healthcare system alone produces four million tonnes of waste annually, which is second only to the food industry [6]. Surgery evidently is a resource and energy-intensive healthcare activity that produces overwhelming quantities of waste [7]. How can surgical practices as seen in high-income countries (HICs) be delivered to the nascent field of global surgery in LMICs, without compromising operating standards and outcomes whilst being environmentally sustainable?

One elegant approach to reducing the impact of surgery on the environment is underpinned by the 5Rs: *Reduce*, *Reuse*, *Recycle*, *Rethink,* and *Research *[8]. Efficient waste reduction strategies combined with reducing the carbon footprint of the operating room have the added benefit of lowering healthcare costs [9]. Global surgery must integrate the 5R strategy to become a robust surgical system in LMICs that is scalable, whilst providing equitable surgical care to the world’s population based on the tenets of access, need, and quality [5].

## Review

Reduce

Reducing energy consumption is one of the three key components (energy, travel, and supply chain) to achieving net zero emissions in the provision of essential healthcare. Common and inexpensive HIC technologies when transferred to LMICs can target energy demands, with little to no adaptations. For example, surgical theatres are flooded with bright and highly efficient LEDs. The LEDs are both 85% less expensive to run and produce 85% less carbon dioxide (CO2) than their filament-based predecessors [10]. The LEDs do not use mercury unlike fluorescent lights; mercury is difficult to handle safely and damaging to ecosystems [10]. Furthermore, LEDs emit less ultraviolet (UV) light (UV light attracts insects) thereby reducing transmission of vector-borne diseases which commonly plague LMICs. Such a ‘small’ intervention can have such far outreaching effects, benefitting the environment whilst enhancing patient safety and care [10].

Reducing travel, thereby altering the supply chain is crucial both environmentally and economically. The LMIC governments can reduce CO2 emissions by investing in favorable geographical sourcing, logistics, and eco-conscious suppliers thus limiting travel distance and reducing unnecessary packaging of goods. [11]. Investing in companies with an ethos in decarbonizing stimulates economic growth, creates employment, reduces pollution, and significantly improves the wider determinants of health such as lifestyle, economic stability, and education. [11].

Reuse

Globally, surgery must endeavor to locally source surgical equipment, reusing and reprocessing instruments where possible without compromising infection control. The World Health Organisation (WHO) estimates that of the 16 billion injections given worldwide per annum, 40% are given using reused and unsterilized needles, which is overwhelmingly a practice in LMICs [12]. This equates to a directly relatable 1.3 million deaths and 26 million years of life lost from blood-borne diseases such as hepatitis B, C, and human immunodeficiency virus (HIV) [13]. It is estimated that regulated ‘buy-back’ schemes of reprocessed medical devices can lead to a 50% reduction in equipment cost, consequently leading to a reduction in the spread of the aforementioned communicable diseases [14]. An elegant example is reusable surgical linens (2% of all hospital waste) such as gowns and drapes [15]. One medical center reported avoiding close to 63,000kg of waste whilst simultaneously saving $38,000 in transport costs over one year with the use of reusable surgical linens. Surgeons report a preference for reusable products over single-use products on the grounds of increased comfort, ease of use, and superior protective properties [16]. Redistributing finances and investing in sterilization equipment leads to a reduction of waste and energy usage that helps save on unnecessary costs for LMICs.

Recycle

Infectious/hazardous operating room (OR) waste totals 10% to 25% of the waste produced by operating theatres [17]. Hazardous waste is most commonly incinerated, releasing carcinogens and greenhouse gases, and consuming considerably more energy than non-hazardous waste disposal [18]. The OR waste can be judiciously recycled, if not soiled with potentially infectious matter such as bodily fluids. Up to 90% of the waste is placed in inappropriate bags and is therefore over-processed to not only release greenhouse gases but also avoid being recycled [8]. Improper segregation of waste allows communicable diseases such as typhoid, cholera, hepatitis, and HIV to find their way into ‘non-hazardously’ labeled waste bags [19]. Developing countries lacking sound surgical waste management/recycling systems precipitate ‘informal recycling’ whereby syringes and needles are collected and sold back to in-need medical communities propagating the outbreak of dangerous diseases [20]. Educating theatre staff in basic OR recycling etiquette is a simple and inexpensive measure that is attainable globally [21]. Safe disposal, proper segregation, and adequate recycling of surgical waste reduce the number of avoidable patient-to-patient transmissions whilst being environmentally sustainable. 

Rethink 

A global attitude of rethinking how surgical care is provided is necessary at departmental, institutional and national levels. ‘Surgathons’ (surgery hackathons) promote innovation and work with the local community's resources to solve problems. Surgathons help rethink and reimagine established practices, often with the use of seemingly non-surgical items. This leads to local surgical innovation, providing an ecosystem that is both ethical and sustainable. This is not only beneficial from a medical perspective but allows local financial investment and feeds back into local economies [22]. Locally sourced and manufactured surgical supplies, prosthetics and drugs are key to alleviating the environmental burden of global surgery. Local manufacturing not only reduces cost but also carbon footprint [22]. One well-documented example is the use of sterilized mosquito nets for hernia mesh repair. This ad hoc and innovative surgical solution show no significant difference in clinical outcomes when compared to standard surgical mesh. In addition to surgical materials, rethinking collaborative surgical training on an international scale will form part of the solution. 'Twinning' between HIC hospitals and their respective surgeons with the LMIC counterparts will allow the transfer of knowledge and technology, skills, service provision, and research [23]. Twinning incubates mutually-beneficial learning, allowing for incremental iterations and betterment in sustainable surgical practices. The LMIC-centred research and appropriate scientific documentation are needed to raise awareness about inventing locally relevant protocols and guidelines on using the available resources [24].

Research

Latin America, Africa, and India contribute to 42% of the world’s population but represent only 7.9% of the research output and LMICs in total contribute only 4% of the medical literature [25]. Inherent systemic barriers are most commonly cited for low surgical research output such as lack of sustainable funding, lack of research training, lack of a culture of research, and issues with record keeping and data management [26]. How can we encourage the practice of evidence-based medicine in LMICs with such a disparate absence of data [5]? The LMIC and HIC scientific communities must work as partners to close the gap in research output. There are gaping holes in data from LMICs leading to an insufficient drive in national resource and fund allocation [25]. A bottom-up approach of increasing data and research will not only benefit patient care directly but have a profound impact on the environment. Medical supplies will reach the in-need places, waste will be reduced and the entire process becomes more cost-effective. 

In HICs, cataract surgery is the most expensive and highest-volume surgery in the entirety of medicine [27]. In the UK a single cataract procedure produces an estimated 180kg carbon dioxide equivalents (CO2e), whilst the same surgery with similar outcomes in India produces 6kg CO2e (5% compared to the UK) [28,29]. Cataract surgery is a compelling example of how LMICs can produce similar safety profiles and comparable outcomes whilst using considerably less energy and fewer resources [30]. The HICs have much to learn from low-cost, sustainable solutions.

## Conclusions

The 5R strategy only provides a framework on which to build solutions to reduce the effects of surgery on the environment. Global surgery must respond, innovate, and progress in the concerted response to tackle climate change. Evidently, surgery is one of the most resource-intensive and highest environmental-impact areas of a hospital. All the limitations and challenges that surgery poses on the environment must be seen as an opportunity to improve. The opportunities arising from the limitations must benefit patient care and not hinder it. Whilst the future must progress in the direction of forming mutually beneficial partnerships between HICs and LMICs, it is imperative that global surgery nurture a symbiotic relationship between its practices and the environment. With adequate international collaboration and awareness of the lack of necessary surgical interventions in LMICs, global surgery has the potential to reduce the impact of surgical practice on the environment, without compromising patient safety or quality of care.
